# Regional dietary patterns and their impact on maternal, placental, and fetal fatty acid profiles during pregnancy: a multicenter study from Croatia

**DOI:** 10.3389/fnut.2026.1883073

**Published:** 2026-08-05

**Authors:** Mislav Herman, Marina Ivanisevic, Josip Delmis

**Affiliations:** Referral Centre Ministry of Health, Department of Obstetrics and Gynecology, University Hospital Centre Zagreb, School of Medicine University of Zagreb, Zagreb, Croatia

**Keywords:** DHA, fatty acids, fetal programming, maternal nutrition, Mediterranean diet, placenta, pregnancy

## Abstract

**Background:**

Maternal dietary fat quality during pregnancy plays a crucial role in fetal growth, neurodevelopment, and long-term metabolic health. Regional dietary patterns may influence maternal–fetal fatty acid transfer, yet data integrating maternal, placental, and fetal compartments remain limited. This study evaluates the impact of regional dietary patterns on fatty acid composition in maternal venous serum, placental tissue, and umbilical venous serum in pregnant women from three distinct regions of Croatia.

**Methods:**

This multicenter prospective study included 44 healthy pregnant women undergoing elective cesarean delivery in Osijek (Slavonia), Zagreb (Central Croatia), and Split (Dalmatia) during the study period (2014–2017). Fatty acid composition was analyzed in maternal venous serum, placental tissue, and umbilical venous serum using gas chromatography. Saturated, monounsaturated, and polyunsaturated fatty acids (n-3 and n-6) were quantified and compared across regions. The primary statistical methods used Kruskal-Wallis test with Bonferroni correction and Spearman's rank correlation.

**Results:**

Maternal venous serum DHA concentrations and proportions were significantly higher in women from Split and Zagreb compared with Osijek. The AA:DHA ratio was highest in the Osijek group and lowest in Split, reflecting regional dietary differences. Fatty acid concentrations of total fatty serum in umbilical venous serum were strongly correlated with maternal serum levels (ρ = 0.71, *P* < 0.001) but were significantly lower in absolute terms. Despite this, the fetus exhibited higher relative proportions of DHA and arachidonic acid and lower proportions of linoleic acid, indicating selective placental transfer. Placental DHA proportion was highest in the Split group, while placental arachidonic acid concentration was highest in Osijek.

**Conclusions:**

Regional dietary patterns significantly influence maternal, placental, and fetal fatty acid composition. Diets rich in marine foods are associated with more favorable DHA status and AA:DHA ratios during pregnancy. These findings support targeted nutritional counseling and DHA/EPA supplementation in regions with traditionally low marine food intake.

## 1 Introduction

Maternal nutrition during pregnancy is a critical determinant of fetal growth, neurodevelopment, and long-term cardiometabolic health. Among dietary components, the quality and balance of dietary fats—particularly long-chain polyunsaturated fatty acids (LC-PUFAs)—have received increasing attention due to their essential roles in placental function, fetal brain development, and metabolic programming (1–3). Docosahexaenoic acid (DHA) and arachidonic acid (AA) are selectively transferred from the mother to the fetus via the placenta, where they accumulate preferentially in neural and retinal tissues. In contrast, excessive intake of saturated fatty acids or an imbalanced n-6: n-3 ratio has been associated with adverse pregnancy outcomes and long-term disease risk in offspring (4–6). Despite international recommendations emphasizing adequate DHA intake during pregnancy, population adherence remains highly variable and is strongly influenced by regional dietary habits.

The intrauterine origin of chronic diseases in adulthood is rooted in Barker's hypothesis of fetal programming, first presented approximately six decades ago, which opened an entirely new era of research. This hypothesis is based on Barker's original observation that fetal undernutrition represents a fundamental cause of ischemic heart disease in adulthood among the population of England and Wales (7). According to this hypothesis, the origins of chronic diseases in adulthood can be traced back to the earliest stages of human development, namely the fetal period. Barker proposed that intrauterine environmental factors induce epigenetic modifications that potentially irreversibly alter the expression of inherited genes without affecting the nucleotide sequence of deoxyribonucleic acid (DNA) (8). In this way, Barker primarily attributed the origins of numerous chronic diseases of childhood and adulthood to the intrauterine environment.

A correlation has been observed between disturbances in intrauterine nutritional status and the development of numerous chronic diseases, particularly type 2 diabetes mellitus, hypertension, chronic kidney disease, coronary heart disease, dyslipidemia, metabolic syndrome, bronchial asthma, malignant diseases, as well as certain psychiatric disorders (schizophrenia, depression) (8, 9).

**Figure 1:**
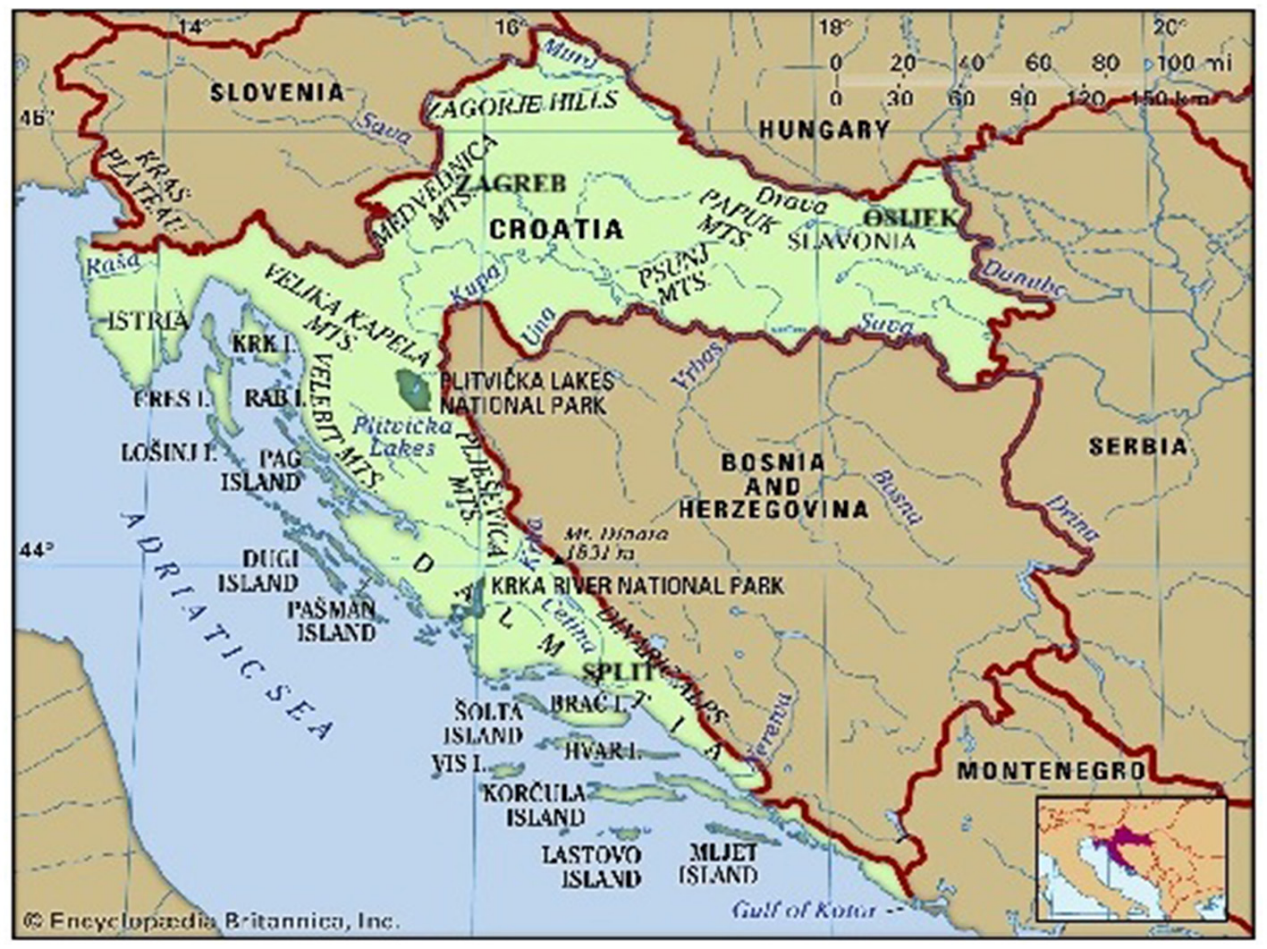
Map of Croatia.

Due to its turbulent history and geopolitical position, the Republic of Croatia has been exposed to various cultural influences, which have also shaped the dietary habits of its population and its culinary tradition. Croatian cuisine is characterized by marked heterogeneity, which is why it is often referred to as a regional cuisine. Despite the country's relatively small geographical area, each region has its own distinct culinary heritage (Figure 1).

Counties located along the Adriatic coast are under strong culinary influence of classical Mediterranean cuisine, primarily Italian and French. Northern counties, including the City of Zagreb and its surroundings, exhibit characteristics of Hungarian, Austrian, and German cuisine, whereas eastern counties show a pronounced influence of Turkish and Hungarian culinary traditions.

The geographical region of Slavonia and Baranja is characterized by highly fertile soil, dense lowland forests, and the calm flows of the major rivers Sava, Drava, and Danube. These conditions favored the development of agriculture and extensive livestock breeding, as well as a diet that is considerably more abundant than in other regions of Croatia. From a culinary perspective, this diet is closely related to the neighboring northern Pannonian region, which is reflected in the use of strong and spicy seasonings, particularly hot red paprika and chili peppers. At the same time, the legacy of former Turkish rule cannot be overlooked, as it introduced and established numerous Eastern Mediterranean fruits, vegetables, and culinary techniques in this area.

Slavonia and Baranja are well known for a specific breed of black, fatty pigs raised in large herds, which explains the prominent role of pork and its products (sausages, blood sausages, smoked ham, bacon, cracklings, kulen, head cheese) in the local diet. In addition to pork, dishes based on beef and veal, poultry, and eggs are also common. In areas along major rivers, especially in the Danube region, freshwater fish dishes play an important role, with catfish, carp, pike-perch, and pike being the most common species. Fish is prepared by boiling, frying in lard, roasting on a spit or grill, as well as drying in the sun or smoke.

Dairy products are primarily based on cow's milk and include cheese, cream, and butter. Various types of vegetables—fresh, cooked, or pickled—are also widely consumed. The grain-producing capacity of Slavonia is reflected in the preparation of numerous dishes made from wheat or corn flour, particularly bread and pastries.

The impact of traditional Slavonian cuisine on health can be examined from the perspective of cardiovascular diseases, which represent the leading cause of death and disability in the Croatian population and are closely associated with dietary habits. From the standpoint of perinatal medicine, a maternal diet rich in saturated fatty acids and the resulting obesity are associated with preeclampsia, preterm birth, increased umbilical artery resistance, as well as obesity and impaired lipid metabolism in the offspring (10, 11).

Dalmatian cuisine follows the principles of modern nutritional recommendations. Light cooking methods such as boiling and grilling, a high intake of fish, olive oil, vegetables, and wild coastal herbs are the main reasons why this cuisine is considered particularly healthy. Dalmatian wines, olive oil, and olives have been valued since ancient times. Although each area still prepares certain dishes in its own unique way, Dalmatian cuisine represents a distinct culinary world whose characteristics have only recently been fully recognized. Based on its components and characteristics, it is classified as a subtype of the Mediterranean diet.

In recent years, the Mediterranean diet has been recognized as the gold standard of healthy nutrition, with numerous long-term beneficial effects on health (11). The Mediterranean diet is characterized as a liberal vegetarian diet, rich in n-3 fatty acids, dietary fiber, B-group vitamins, and various antioxidants, while being low in saturated fats.

The Mediterranean diet was defined as a dietary pattern rich in minimally processed plant-based foods, fresh fruits and nuts, freshly caught fish and seafood, with small amounts of dairy products primarily in the form of cheese and yogurt. Olive oil is used as the main source of fat. Red meat is consumed only occasionally and in small quantities, eggs less than four times per week, and meals are traditionally accompanied by small to moderate amounts of red wine (12).

Fish, as a key component of the Mediterranean diet, is an exceptionally healthy food, low in saturated fatty acids and rich in proteins, polyunsaturated fatty acids, and other essential nutrients (iodine, vitamin D, selenium). Most importantly, it represents the primary source of two long-chain n-3 polyunsaturated fatty acids: docosahexaenoic acid (DHA) and eicosapentaenoic acid (EPA) (13).

The diet of the City of Zagreb and its surroundings, both in terms of ingredients and cultural habits, is considered a combination of Slavonian and Mediterranean dietary patterns.

**The aim of this study** was to evaluate regional differences in fatty acid composition in maternal venous serum, placental tissue, and umbilical venous and arterial serum in pregnant women from three distinct regions of Croatia. By integrating maternal, placental, and fetal compartments, this study seeks to clarify how maternal diet influences placental fatty acid transfer and fetal exposure during pregnancy.

## 2 Materials and methods

### 2.1 Ethical statement

The Ethics Committee at the Department of Obstetrics and Gynecology (No.3-14) and the School of Medicine's Ethics Committee, the University of Zagreb (380-59-10106-14-4174) approved the study.

### 2.2 Study participants

This multicenter prospective study included three groups of pregnant women from three geographical regions of the Republic of Croatia: Central Croatia, Slavonia, and Dalmatia. Participants were monitored at three university hospital centers: pregnant women from Central Croatia, including the City of Zagreb and its surroundings, at the University Hospital Centre Zagreb; women from Slavonia at the University Hospital Centre Osijek; and women from Dalmatia at the University Hospital Centre Split.

During 2014–2017, a total of 50 pregnant women were enrolled in the study (17 from Zagreb, 17 from Osijek, and 16 from Split). 6 pregnant women were excluded from the study, one from Osijek due to a hypotrophic newborn (2,470 grams), one from Zagreb due to fetal macrosomia 4,400 grams, and 4 from Split 2 due to macrosomia (4,280 grams, 4,170 grams) and two dues to a hypotrophic child (2,570 and 2,610 grams). A total of 44 pregnant women, their placentas, and newborns were included in the study. Participants were enrolled during preoperative assessment for delivery by elective cesarean section at one of the three university hospital centers. Indications for elective cesarean section included previous cesarean delivery or breech presentation. All pregnancies were completed between 38 and 41 weeks of gestation.

#### 2.2.1 Inclusion criteria

Gestational age was determined based on the first day of the last menstrual period and confirmed by first-trimester ultrasound assessment of crown–rump length (between 6 and 10 weeks of gestation).

To avoid the influence of pregnancy-related pathological conditions on lipid status in the mother, fetus, and placenta, samples were collected exclusively from healthy pregnant women with normal body mass index (BMI), normal oral glucose tolerance test results (OGTT), and an uncomplicated course of pregnancy. Due to the physiological increase in arachidonic acid concentrations during vaginal delivery, related to prostaglandin synthesis that initiates and promotes uterine contractions, only pregnancies completed by elective cesarean section without the onset of labor were included. Women undergoing emergency cesarean section after attempted vaginal delivery were excluded.

#### 2.2.2 Exclusion criteria

Exclusion criteria included maternal and pre-existing hypertension, any form of diabetes mellitus, fetal chromosomal abnormalities or malformation syndromes, fetal growth restriction, fetal macrosomia, multiple pregnancy, preterm delivery, or other pathological conditions. Pregnant women on lipid-lowering therapy were excluded. They did not take dietary supplements such as n-3 fatty acids. Prenatal multivitamin use beyond n-3 fatty acid supplements was not systematically assessed and cannot be excluded as a potential source of confounding, particularly for fat-soluble vitamins that may influence fatty acid metabolism.

All participants received verbal information about the study, and detailed written information was provided in the informed consent documents (“Participant Information Sheet” and “Informed Consent for Adult Participation in Research”).

For each participant, three samples were collected for the purposes of the study: 1. maternal venous blood, 2. placental tissue from the central region of the placenta, 3. umbilical venous blood.

### 2.3 Chemicals and materials

Gas chromatography standards (Supelco FAME MIX 37, PUFA No.1, PUFA No.3) were purchased from Supelco Inc., Bellefonte, PA, USA. Organic solvents used for total lipid extraction were obtained from Sigma-Aldrich Chemie GmbH (Steinheim, Germany), as were the internal standard heptadecanoic acid, anhydrous sodium sulfate, 2,6-di-tert-butyl-4-methylphenol (BHT), and concentrated hydrochloric acid.

### 2.4 Blood and tissue sampling

Maternal venous blood samples were collected on the day of delivery in the morning, immediately before the elective cesarean section, after overnight fasting, from the left cubital vein into serum separator tubes (Vacuette^®^ tube 5 mL Z Serum Separator Clot Activator). Within 1 hour of collection, samples were centrifuged at 1,400 × g (3,000 rpm) for 10 min. Serum was separated, aliquoted into two cryogenic tubes, and stored at −70 °C until further analysis.

Umbilical venous samples were collected intraoperatively immediately after delivery of the newborn, while the placenta was still *in situ* in the uterine wall and before administration of uterotonic agents. The *in-situ* approach prevented collapse of umbilical vein, which can occur immediately after placental separation and complicate blood collection. Blood samples were collected into serum separator tubes (Vacuette^®^ tube 5 mL Z Serum Separator Clot Activator), centrifuged within 1 hour at 1,400 × g for 10 min, after which serum was separated, aliquoted into two cryogenic tubes, and stored at −70 °C until analysis.

After placental delivery, samples of chorionic tissue were collected from the central region of the placenta. Using a scalpel on a granite surface, chorionic tissue was separated from capillaries and decidua. The purified chorionic tissue was then rinsed with Ringer's lactate solution through a platinum mesh to remove residual blood and clots and stored in cryogenic tubes at −70 °C until further analysis.

### 2.5 Extraction of total lipids from serum and placental tissue

Extraction of total lipids and conversion of fatty acids into methyl esters were performed at the Department of Chemistry and Biochemistry, School of Medicine, University of Zagreb.

Total lipids from serum samples were extracted using a modified Folch method (13) with organic solvents. Thawed serum samples (2 mL) were mixed with 8 mL of chloroform–methanol (CHCl3: MeOH, 2:1, v/v) containing the antioxidant butylated hydroxytoluene (BHT) and shaken for 10 min. Samples were then centrifuged at 1,400 × g for 5 min. The lower organic phase was transferred into a new glass tube using a Pasteur pipette. The extraction was repeated with an additional 1 mL of solvent mixture. Anhydrous sodium sulfate (Na_2_SO4) was added to bind residual water. The sample was filtered into pre-weighed tubes and evaporated to dryness under vacuum using a UNIVAPO 100H evaporator equipped with a cooling unit (UNICRYO MC2L −80 °C, UniEquip, Martinsried, Germany). Extracted lipids were used for subsequent fatty acid composition analysis.

Placental tissue samples were prepared by homogenizing 1 g of tissue in 3 mL of KCl buffer under cooling, in three 20 s cycles with 10 s cooling intervals, until complete homogenization.

Lipid extraction from placental homogenates was performed using chloroform–methanol mixtures with added BHT. Thawed homogenates were extracted sequentially using CHCl3: MeOH mixtures (2:1, 1:1, and 1:2, v/v), with shaking for 30 min and centrifugation at 2,000 rpm for 5 min after each step. Organic phases were collected into Erlenmeyer flasks. Anhydrous Na_2_SO4 was added to remove residual water, and samples were stored overnight at +4 °C. The following day, samples were filtered into pre-weighed tubes and evaporated to dryness under vacuum using a UNIVAPO 100H evaporator with a UNICRYO MC2L cooling unit (−80 °C). Extracted lipids were used for fatty acid analysis.

Four lipid fractions were isolated and quantified in each of the three biological compartments examined: maternal venous serum, fetal umbilical venous serum, and placental tissue. The fractions comprised cholesterol esters, phospholipids, triglycerides, and non-esterified fatty acids, separated by sequential elution from aminopropyl-bonded silica SPE columns. Total lipid content for each compartment was calculated as the arithmetic sum of the four individual fractions, enabling both compartment-specific and inter-compartmental comparison of lipid distribution (Supplementary Tables S1, S2).

### 2.6 Preparation of fatty acyl methyl esters

Fatty acids were converted into volatile derivatives by transesterification using methanolic HCl, producing fatty acid methyl esters (FAME), according to the international standard ISO 5509 (ISO, 2000).

To the dried lipid extract, 2 mL of methanolic HCl (1 M HCl in methanol) was added. Tubes were sealed and incubated at 100 °C for 45 min. After cooling, FAMEs were extracted with petroleum ether. Five milliliters of petroleum ether were added, the mixture was shaken, centrifuged, and the upper organic layer transferred to a new glass tube. This procedure was repeated four times to ensure complete extraction. The organic phase was washed repeatedly with 5 mL of distilled water until neutral pH was achieved. After discarding the aqueous phase, anhydrous Na_2_SO4 was added overnight to remove residual water. The sulfate was removed by filtration, and the solvent was evaporated to dryness. Samples were flushed with nitrogen, sealed, and stored at −70 °C until gas chromatographic analysis.

### 2.7 Gas chromatography

Gas Chromatography identified 26 individual fatty acids. We report 10 fatty acids selected based on (1) biological relevance to fetal neurodevelopment (DHA, AA, EPA), (2) representation of major lipid classes, and (3) consistent detectability above the limit of quantification. Total fatty acid concentration (μg/mL) was calculated as the sum of all 26 quantified fatty acids. Relative proportions (%) were calculated by dividing individual concentration by total concentration.

Formal LOD and LOQ were not determined as part of the original validation protocol. However, all reported fatty acid concentration were derived from well-defined chromatographic peaks with signal-to-noise ratio exceeding 10. Fatty acids present below reliable quantification were reported as trace amounts in the tables.

Fatty acid methyl esters were analyzed using gas chromatography on an Agilent 7890B system (Agilent Technologies, CA, USA) equipped with a flame ionization detector (FID). Separation was performed on an HP-88 capillary column (100 m × 0.25 mm internal diameter × 0.20 μm film thickness; Agilent J&W, USA). The instrument was equipped with an autosampler and a split/spitless injection system.

Injector temperature was set at 200 C and detector temperature at 250 °C. The initial column temperature was 140 °C for 5 min, followed by a temperature increase of 4 °C/min to 240 °C, which was maintained for 15 min. A sample volume of 0.5 μL was injected in split mode at a ratio of 1:100. Helium (purity 6.0) was used as the carrier gas at a flow rate of 0.8 mL/min.

Individual fatty acids were identified based on retention times by comparison with known standards (PUFA No.1, PUFA No.3, C8–C20, Supelco 37 Component FAME Mix; all Supelco, USA).

Fatty acid content was expressed as relative mass percentage (%) and absolute concentration (μg/mL). Quantification was performed by comparing the peak area of each fatty acid with that of the internal standard (heptadecanoic acid, C17:0; Sigma-Aldrich, Germany). Before transesterification, heptadecanoic acid was added to the dried lipid, enabling correction for variability in methylation efficiency and FAME recovery.

Chromatograms were processed using Agilent 7890B GC software (Agilent Technologies, CA, USA).

### 2.8 Statistical analyses

The sample size was determined by recruitment feasibility constraints inherent to this study design. Simultaneous collection of matched maternal venous blood, umbilical cord blood, and placental tissue from elective cesarean deliveries requires strict eligibility criteria–including normal BMI, normal OGTT, absence of any pregnancy-related pathology, no lipid-lowering therapy or n-3 supplements, and elective (no emergency cesarean section)–that substantially reduce the eligible pool. No *a priori* power calculation was performed, as this was an exploratory study. *Post-hoc* power analysis confirmed >95% power for the primary outcome (DHA proportion, Cohen's d = 1.8), however, the study may have been underpowered to detect smaller but clinically meaningful differences in other fatty acids. The unequal group sizes may affect the precision of between-group comparisons, particularly for the split group (*n* = 12). These limitations underscore the hypothesis generating nature of the findings.

Categorical variables were presented as absolute and relative frequencies. Quantitative variables were described using mean and standard deviation for normally distributed data, and median with interquartile range (25th−75th percentile) for non-normally distributed data.

Normality of distribution was assessed using the Shapiro–Wilk test. Differences between two independent groups were analyzed using Student's *t*-test for normally distributed variables and the Kruskal-Wallis test with Bonferroni correction for pairwise comparisons for non-normally distributed variables. Given the exploratory nature of the study and the large number of fatty acids examined across three biological compartments, the possibility of false-positive findings cannot be excluded despite Bonferroni correction for pairwise comparisons. Findings should be interpreted in the context of the overall pattern of results across compartments rather than relying on individual *P*-values in isolation. Statistically significant findings that are also biologically coherent across compartment (e.g., consistently lower DHA in Osijek across maternal serum, placental tissue, and umbilical cord blood are more likely to reflect true regional differences. Differences in categorical variables were assessed using the χ^2^ test or Fisher's exact test when appropriate. Associations between normally distributed numerical variables were evaluated using Pearson's correlation coefficient (*r*), while Spearman's rank correlation coefficient (ρ) was used for non-normally distributed variables.

All *P* values were two-sided. Statistical significance was set at α = 0.05. Data were analyzed using SPSS software version 25 (IBM Corporation, Chicago, IL, USA).

## 3 Results

Table 1 presents and compares the demographic and clinical characteristics of the studied groups of pregnant women. No significant differences were observed among the groups with respect to maternal age, body mass index, gestational weight gain, or duration of pregnancy. Similarly, neonatal outcomes—including birth weight, birth length, and Apgar scores at 1 and 5 min—did not differ significantly between the groups. Data are presented as median and interquartile range (IQR). Group comparisons were performed using the Kruskal-Wallis test, which revealed no statistically significant differences (*P* > 0.05). In addition, the χ^2^ test showed no significant differences in neonatal sex distribution among the groups (*P* > 0.05).

**Table 1:** Demographic characteristics of the studied groups.

The studied groups	Osijek (*n* = 16) median and IQR; *n* (%)	Zagreb (*n* = 16) median and IQR; *n* (%)	Split (*n* = 12) median and IQR; *n* (%)
Maternal characteristics			
Age (year)	32 (28–35)	31 (29–36)	30 (27–34)
Body mass index before pregnancy (kg/m^2^)	22.4 (21.2–23.6)	23.2 (22.0–24.3)	22.8 (21.0–24.6)
Weight gain during pregnancy (kg)	18.0 (17.5–20.0)	15.0 (12.1–16.4)	15.3 (14.2–17.8)
Gestational age (weeks)	40.0 (40.0–40.0)	39.0 (38.0–40)	39.0 (40.0–40)
Neonatal characteristics			
Newborn weight (g)	3,540 (3,060–3,750)	3,490 (3,280–3,540)	3,350 (3,220–3,700)
Newborn height (cm)	51.0 (50–52)	51.0 (49–52)	50.0 (49–52)
1 min Apgar score	10 (10–10)	10 (10–10)	10 (10–10)
5 min Apgar score	10 (10–10)	10 (10–10)	10 (10–10)
Sex ratio: male/female *n* (%)	8/8 (50/50)	9/7 (56.3/43.7)	7/8 (46.7/53.3)

Table 2 presents fatty acid concentrations and proportions in maternal venous serum from Osijek, Zagreb, and Split. Total fatty acid concentrations, as well as palmitic, stearic, and total SFA concentrations, did not differ between groups, though SFA proportions showed a borderline difference (*P* = 0.044), with the highest values in Split. MUFA fractions—palmitoleic, oleic, and total MUFA—were likewise comparable across all three sites.

**Table 2:** Concentration of total fatty acids and concentration and proportion of saturated, monounsaturated, and polyunsaturated fatty acids in maternal venous serum.

Fatty acids	Osijek (*n* = 16) median and IQR	Zagreb (*n* = 16) median and IQR	Split (*n* = 12) median and IQR	*P*	^*^P
Total fatty acids (μg/mL)	478.1 (312.6–677.5)	630.4 (295.6–622.1)	622.1 (218.1–801.9)	0.820	
Palmitic acid (C16:0) (μg/mL)	156.2 (125.1–203.4)	181.5 (86.8–207.4)	205.4 (50.6–259.4)	0.678	
Stearic acid (C18:0) (μg/mL)	31.9 (19.9–44.2)	37.4 (22.1–62.0)	41.3 (7.2–48.0)	0.517	
Total saturated fatty acids (μg/mL)	184.7 (154.0–253.6)	265.8 (119.9–345.4)	264.2 (98.1–324.1)	0.154	
Palmitic acid (C16:0) (weight %)	31.3 (30.8–32.6)	31.3 (29.0–32.3)	31.2 (27.0–36.0)	0.976	
Stearic acid (C18:0) (weight %)	6.5 (6.2–7.0)	5.7 (5.1–7.0)	6.3 (5.8–7.3)	0.523	
Saturated fatty acids (weight %)	38.4 (36.0–39.6)	40.4 (38.1–41.4)	41.8 (38.0–43.7)	0.044	
Palmitoleic acid (C16:1 n-7) (μg/mL)	5.5 (0.0–13.2)	14.0 (6.3–24.3)	17.1 (21.3–24.8)	0.063	
Oleic acid (C18:1 n-9 cis) (μg/mL)	95.2 (69.5–134.0)	87.5 (49,9–139.1)	118.3 (22.3–189.1)	0.346	
Elaidic acid (C:18:1n-9 trans) (μg/mL)	0.0 (0.0–11.2)	13.1^a^ (3.0–19.3)	0.0^a^ (0.0–3.7)	0.022	0.048^a^
Total monounsaturated fatty acids (μg/mL)	111.2 (87.1–164.8)	125.5 (63.3–186.9)	162.8 (29.9–213.0)	0.717	
Palmitoleic acid (C16:1 n-7) (weight %)	1.4 (0.3–2.0)	2.6 (2.0–2.8)	2.5 (2.0–2.9)	0.390	
Oleic acid (C18:1 n-9) (weight %)	18.5 (14.6–19.8)	16.8 (13.8–19.0)	21.0 (16.5–22.7)	0.346	
Elaidic acid (C:18:1n-9 trans) (weight %)	0.0 (0.0–2.5)	2.3 (0.9–2.6)	0 (0.0–1.9)	0.086	
Total monounsaturated fatty acids (weight %)	20.1 (16.5–23.8)	21.9 (18.4–25.1)	23.7 (21.1–26.1)	0.827	
α-linolenic acid (C18:3 n-3) (μg/mL)	0.3 (0.0–1.0)	0.9 (0.6–1.0)	0.8 (0.0–2.7)	0.578	
Eicosapentaenoic acid (C20:5 n-3) (μg/mL)	0.003 (0.0–0.08)	0,06 (0.0–0.1)	0.0 (0.0–0.2)	0.619	
Docosahexaenoic acid (C22:6 n-3) (μg/mL)	6.4^ab^ (3.5–10.9)	16.1^a^ (12.1–22.6)	17.9^b^ (16.3–23.8)	0.002	0.014^a^ 0.002^b^
Total polyunsaturated n-3 fatty acids (μg/mL)	7.3^ab^ (3.5–15.2)	19.5^a^ (13.2–25.5)	20.5^b^ (17.3–30.9)	0.001	0.017^a^ 0.004^b^
α-linolenic acid (C18:3 n-3) (weight %)	0.09 (0.0–0.2)	0.1 (0.0–0.3)	0.0 (0.0–0.1)	0.586	
Eicosapentaenoic acid (C20:5 n-3) (weight %)	0.05 (0.0–0.09)	0.07 (0.0–0.1)	0.09 (0.0–0.1)	0.610	
Docosahexaenoic acid (C22:6 n-3) (weight %)	1.3^ab^ (1.1–2.0)	2.2^a^ (1.5–3.8)	3.6^b^ (2.2–4.4)	0.001	0.034 0.004^b^
Total polyunsaturated n-3 fatty acids (weight %)	1.9^a^ (1.3–2.1)	2.5^b^ (2.0–3.8)	3.9^ab^ (2.2–5.9)	0.001	0.024^a^ 0.001^b^
Linoleic acid (C18:2 n-6) (μg/mL)	140.7 (84.1–188.3)	136.3 (67.1–229.8)	155.8 (21.7–207.0)	0.530	
γ-linolenic acid (C18:3 n-6) (μg/mL)	0.1 (0.0–0.2)	0.1 (0.0–0.1)	0.0 (0.0–0.19)	0.419	
Arachidonic acid (C20:4 n-6) (μg/mL)	31.1 (27.4–42.0)	35.5 (19.2–54.8)	36.2 (16.8–48.6)	0.748	
Total polyunsaturated n-6 fatty acids (μg/mL)	179.5 (104.8–245.3)	181.7 (94.4–287.1)	207.0 (35.7–259.2)	0.799	
Linoleic acid (C18:2 n-6) (weight %)	28.0 (25.4–28.7)	25.1 (21.0–27.1)	24.9 (21.1–28.9)	0.442	
γ-linolenic acid (C18:3 n-6) (weight %)	0.1 (0.0–0.2)	0.1 (0.0–0.1)	0.0 (0.0–0.1)	0.519	
Arachidonic acid (C20:4 n-6) (weight %)	7.1 (6.2–8.1)	5.4 (3.6–6.7)	6.0 (4.4–6.6)	0.746	
Total polyunsaturated n-6 fatty acids (weight %)	36.2^a^ (33.5–38.7)	31.9 (29.2–35.3)	31.2^a^ (27.1–35.2)	0.035	0.048^a^
AA/DHA (weight %)	4.7^ab^ (3.9–5.6)	2.7^a^ (1.5–3.6)	2.0^b^ (1.5–3.1)	< 0.001	0.002^a^ 0.001^b^

Kruskal-Wallis test, ^*^Significance value adjusted by the Bonferroni correction for multiple tests. ^ab^Show statistical significance between the studied groups.

Elaidic acid was notably elevated in Zagreb compared to both Split (*P* = 0.048). Among n-3 fatty acids, α-linolenic and EPA showed no significant differences in either concentration or proportion. DHA concentration and proportion were highest in Split, differing significantly from Osijek and Zagreb (*P* = 0.004, *P* = 0.034). Total n-3 concentrations followed the same pattern: Split > Zagreb > Osijek, with Split differing significantly from Osijek (*P* = 0.001), and n-3 proportions additionally differing between Split and Zagreb (*P* = 0.003).

Linoleic, γ-linolenic, arachidonic, and total n-6 concentrations did not differ between groups, nor did linoleic and γ-linolenic proportions. However, n-6 proportions were highest in Osijek, significantly exceeding both Split (*P* = 0.048). The AA/DHA ratio was highest in Osijek and significantly lower in both Zagreb and Split (*P* =0.002, *P* = 0.001 for both), with no significant difference between the latter two.

**Figure 2:**
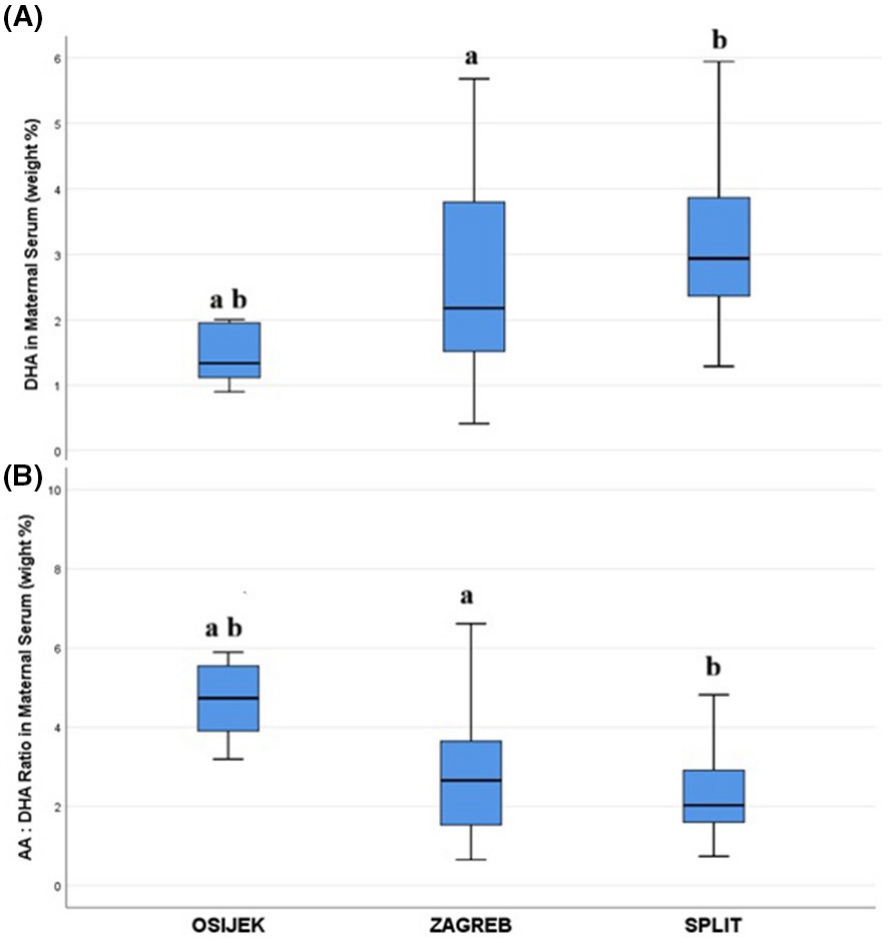
Regional differences in maternal serum DHA proportion and AA: DHA ratio. **(A)** Docosahexaenoic acid (DHA) proportion in maternal venous serum by region. Box plot show median and interquartile range, whiskers extend to minimum, and maximum values. (Osijek (Slavonia, *n* = 16), Zagreb (Central Croatia, *n* = 16), Split (Dalmatia, *n* = 12). Kruskal-Wallis test ^a^*P* = 0.034, ^b^*P* = 0.004). **(B)** Ratio (AA: DHA) by region. Box plot show median and interquartile range, whiskers extend to minimum, and maximum values [Osijek (Slavonia, *n* = 16), Zagreb (Central Croatia, *n*=16), Split (Dalmatia, *n* = 12). Kruskal-Wallis test ^a^*P* = 0.002, ^b^*P* = 0.001].

Figure 2 shows the regional differences in the proportions of DHA and the AA: DHA ratio in maternal venous serum. Pregnant women from the Osijek group have the lowest proportion of DHA and the highest ratio of AA: DHA.

**Figure 3:**
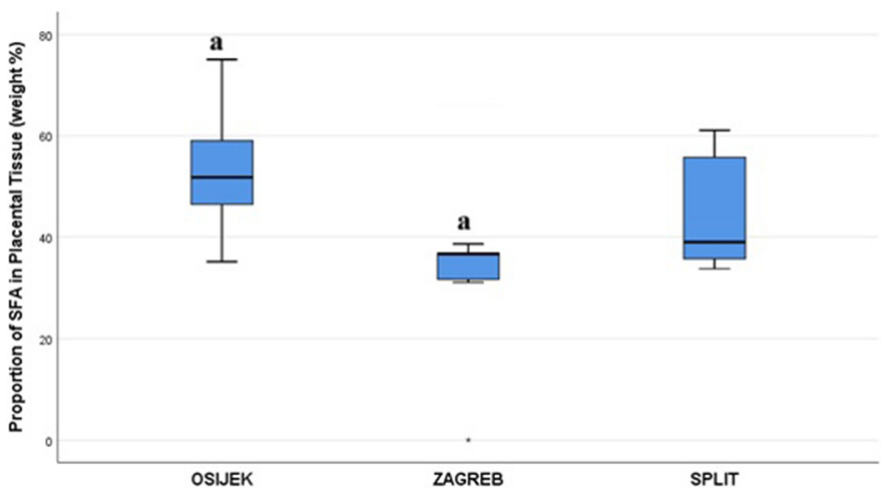
Regional differences in placental tissue SFA proportion. Box plot show median and interquartile range, whiskers extend to minimum, and maximum values [Osijek (Slavonia, *n* = 16), Zagreb (Central Croatia, *n* = 16), Split (Dalmatia, *n* = 12). Kruskal-Wallis test ^a^*P* = 0.006].

Supplementary Table S3 presents fatty acid concentrations and proportions in placental tissue. Total fatty acid, palmitic acid, and stearic acid concentrations did not differ between groups. Total SFA concentrations were highest in Osijek, significantly exceeding Zagreb (*P* = 0.020), but not Split. SFA proportions were also highest in Osijek, significantly above Zagreb (*P* = 0.006), with no other pairwise differences (Figure 3).

Palmitoleic, and oleic, concentrations were all highest in Split, differing significantly from Osijek, and Zagreb (*P* = 0.040, and 0.048), while total MUFA concentration showed no group difference. Elaidic acid concentrations were highest in Zagreb, differing significantly from Osijek (*P* = 0.003). For proportions, oleic acid was highest in Split and lowest in Osijek (*P* = 0.048). Total MUFA proportion was highest in Split, significantly above Osijek (*P* = 0.047) (Supplementary Table S3).

Linoleic acid and γ-linolenic acid concentration did not differ between groups. Arachidonic acid concentration was also highest in Osijek, significantly above Split (*P* = 0.004), and Zagreb (*P* = 0.007). γ-Linolenic and total n-6 concentrations showed no group differences, nor did the proportions of any n-6 fatty acid (Supplementary Table S3).

Table 3 presents fatty acid concentrations and proportions in umbilical venous serum. Total fatty acid concentration was highest in Split, significantly exceeding Osijek (*P* = 0.016), and Zagreb (*P* = 0.048).

**Table 3:** Concentration of total fatty acids and concentration and proportion of saturated, monounsaturated, and polyunsaturated fatty acids in umbilical venous serum.

Fatty acids	Osijek (*n* = 16) median and IQR	Zagreb (*n* = 16) median and IQR	Split (*n* = 12) median and IQR	*P*	^*^P
Total fatty acids (μg/mL)	111.7^ab^ (85.3–170.4)	223.1^b^ (165.6–245.7)	250.5^a^ (199.5–432.7)	0.010	0.016^a^ 0.048^b^
Palmitic acid (C16:0) (μg/mL)	47.4^a^ (32.3–56.0)	61.9 (47.8–72.0)	92.0^a^ (61.7–126.0)	0.032	0.026^a^
Stearic acid (C18:0) (μg/mL)	14.0^a^ (9.0–19.2)	24.9 (17.0–29.6)	33.4^a^ (24.9–39.7)	0.027	0.024^a^
Total saturated fatty acids (μg/mL)	59.5^ab^ (44.3–78.0)	107.0^b^ (82.2–143.8)	132.3^a^ (89.0–177.0)	0.014	0.030^a^ 0.039^b^
Palmitic acid (C16:0) (weight %)	31.0 (15.1–33.4)	27.5^a^ (22.6–29.6)	31.8^a^ (31.2–34.7)	0.003	0.002^a^
Stearic acid (C18:0) (weight %)	10.6 (0.0–12.0)	10.4 (6.8–11.2)	10,7 (9.9–12.0)	0.357	
Total saturated fatty acids (weight %)	44.2 (31.0–45.9)	55.5 (43.3–54.8)	44.5 (43.2–51.8)	0.108	
Palmitoleic acid (C16:1 n-7) (μg/mL)	1.7 (0.0–5.2)	2.9 (0.0–5.9)	12.4 (5.5–17.8)	0.084	
Oleic acid (C18:1 n-9) (μg/mL)	13.3^a^ (4.0–24.1)	31.1 (21.3–33.0)	85.9^a^ (32.0–98.1)	0.006	0.006^a^
Elaidic acid (C:18:1n-9 trans) (μg/mL)	0.0 (0.0–3.4)	0.0 (0.0–5.8)	0.0 (0.0–0.0)	0.465	
Total monounsaturated fatty acids (μg/mL)	18.9^a^ (3.0–32.1)	40.2 (28.7–42.8)	43.6^a^ (35.5–119.9)	0.014	0.015^a^
Palmitoleic acid (C16:1 n-7) (weight %)	2.3 (0.0–3.2)	1.6 (0.0–2.3)	3.6 (2.7–4.3)	0.115	
Oleic acid (C18:1 n-9) (weight %)	13.7 (6.0–15.7)	13.0 (10.8–14.2)	17.4 (14.2–19.7)	0.204	
Elaidic acid (C:18:1n-9 trans) (weight %)	0.0 (0.0–3.2)	0.0 (0.0–2.5)	0.0 (0.0–0.0)	0.356	
Total monounsaturated fatty acids (weight %)	19.9 (0.0–20.8)	17.7 (14.3–19.5)	19.7 (14.8–19.9)	0.342	
α-linolenic acid (C18:3 n-3) (μg/mL)	In trace	In trace	In trace	-	
Eicosapentaenoic acid (C20:5 n-3) (μg/mL)	In trace	In trace	In trace	-	
Docosahexaenoic acid (C22:6 n-3) (μg/mL)	1.8^ab^ (0.0–3.9)	6.1^a^ (2.1–7.6)	5.8^b^ (2.6–7.9)	0.005	0.021^a^ 0.014^b^
Total polyunsaturated n-3 fatty acids (μg/mL)	3.6^a^ (1.4–5.6)	6.9 (3.9–8.9)	14.1^a^ (8.5–19.5)	0.003	0.002^a^
α-linolenic acid (C18:3 n-3) (weight %)	-	-	-	-	
Eicosapentaenoic acid (C20:5 n-3) (weight %)	-	-	-	-	
Docosahexaenoic acid (C22:6 n-3) (weight %)	1.7 (0.0–2.6)	2.1 (1,.3–3.4)	1.9 (1.4–2.5)	0.292	
Total polyunsaturated n-3 fatty acids (weight %)	1.9^ab^ (1.1–4.2)	5.6^a^ (3.6–7.8)	7.3^b^ (4.8–8.9)	0.023	0.043^a^ 0.025^b^
Linoleic acid (C18:2 n-6) (μg/mL)	14.1^a^ (0.0–23.1)	30.5 (21.3–35.8)	37.9^a^ (27.8–53.4)	0.009	0.023
γ-linolenic acid (C18:3 n-6) (μg/mL)	In trace	In trace	In trace	-	
Arachidonic acid (C20:4 n-6) (μg/mL)	15.0^a^ (4.4–28.3)	18.8 (13.6–28.8)	41.6^a^ (20.1–48.9)	0.001	0.019^a^
Total polyunsaturated n-6 fatty acids (μg/mL)	46.8^a^ (32.6–49.9)	51.2 (43.9–61.4)	84.3^a^ (54.5–119.4)	0.002	0.027^a^
Linoleic acid (C18:2 n-6) (weight %)	11.0 (0.0–14.0)	12.5 (10.0–14.5)	11.8 (10.0–15.4)	> 0.9	
γ-linolenic acid (C18:3 n-6) (weight %)	2.2 (0.0–2.7)	0.0 (0.0–1.8)	2.0 (1.8–2.3)	0.394	
Arachidonic acid (C20:4 n-6) (weight %)	14.5^ab^ (12.0–16.5)	9.4^a^ (6.8–10.7)	10.5^b^ (10.5–8.8)	0.001	0.001^a^ 0.031^b^
Total polyunsaturated n-6 fatty acids (weight %)	30.0^a^ (22.8–31.5)	21.2^a^ (19.3–26.7)	29.4 (25.5–31.2)	0.013	0.024^a^
AA/DHA (weight %)	6.9^a^ (5.3–11.7)	3.1^a^ (1.2–6.1)	4.8 (2.3–5.5)	0.023	0.033^a^

Kruskal-Wallis test, ^*^Significance value adjusted by the Bonferroni correction for multiple tests. ^a, b^Show statistical significance between the studied groups.

Among SFAs, palmitic, stearic, and total SFA concentrations were all highest in Split, each significantly above Osijek (*P* = 0.026, 0.024, and 0.030). Total SFA concentrations in Split were higher when compared to Zagreb (*P* = 0.039). Palmitic acid proportion was also highest in Split, significantly exceeding Zagreb (*P* = 0.002). Stearic and total SFA proportions did not differ between groups.

For MUFAs, oleic acid concentrations were both highest in Split, significantly exceeding Osijek (oleic: *P* = 0.006). Total MUFA concentration was highest in Split, significantly above Osijek (*P* = 0.014). Palmitoleic acid, elaidic acid, and total MUFA proportions showed no group differences.

α-Linolenic and EPA were present only in trace amounts and excluded from analysis. DHA and total n-3 concentration was significantly higher in both Zagreb and Split compared to Osijek (*P* = 0.021 and 0.014), with no difference between the former two. Total n-3 concentration followed the same pattern. DHA proportion did not differ significantly between groups, while n-3 proportion was significantly higher in Split than Osijek (*P* = 0.025), and in Split than Zagreb (*P* = 0.025).

Linoleic and arachidonic acid concentrations were highest in Split, both significantly exceeding Osijek (*P* = 0.023, 0.019), without significant differences vs. Zagreb. Total n-6 concentration was also highest in Split, significantly above Osijek (*P* = 0.027). For proportions, arachidonic acid was highest in Osijek, significantly exceeding Zagreb (*P* = 0.001), and n-6 proportion was likewise highest in Osijek, significantly above Zagreb (*P* = 0.024). The AA/DHA ratio was highest in Osijek, significantly exceeding Zagreb (*P* = 0.033), with no difference between Zagreb and Split.

**Figure 4:**
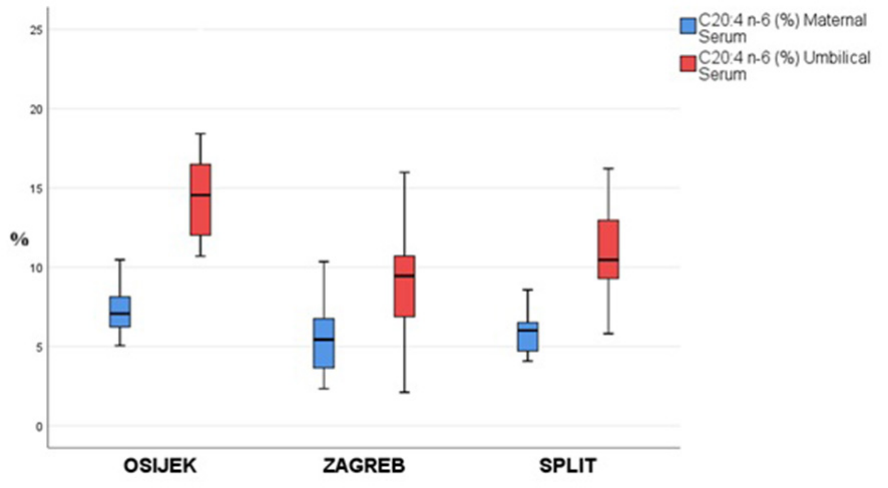
Regional differences in maternal and, umbilical venous sera AA. Box plot show median and interquartile range, whiskers extend to minimum, and maximum values [Osijek (Slavonia, *n* = 16), Zagreb (Central Croatia, *n* = 16), Split (Dalmatia, *n* = 12). Wilcoxon Signed Ranks test: Osijek *P* = 0.001, Zagreb *P* = 0.013, Split *P* = 0.004].

The fetus exhibited significantly higher proportions of arachidonic acid compared with the mother, whereas the mother showed a higher proportion of linoleic acid than the fetus (Figure 4).

Selective placental transfer of long-chain polyunsaturated fatty acids. Comparison of arachidonic acid (AA), (% of total fatty acids) between maternal venous serum (blue bars) and umbilical venous serum (red bars). Data are presented as median values 44 mother-infant pairs. Differences between maternal and fetal compartments were analyzed using Wilcoxon signed-rank test *P* < 0.001. The fetus exhibited significantly higher proportions of AA compared with the mother.

Nonparametric correlation analyses (Spearman's rho) revealed significant positive associations between maternal and umbilical venous serum fatty acid concentrations across all lipid classes. The strongest correlation was observed for n-6 PUFAs (ρ = 0.806, *P* < 0.001), followed by total fatty acids (ρ = 0.801, *P* < 0.001), MUFAs (ρ = 0.735, *P* < 0.001), and n-3 PUFAs (ρ = 0.721, *P* < 0.001). These findings confirm that maternal fatty acid status is a primary determinant of fetal fatty acid availability (Supplementary Table S4).

**Figure 5:**
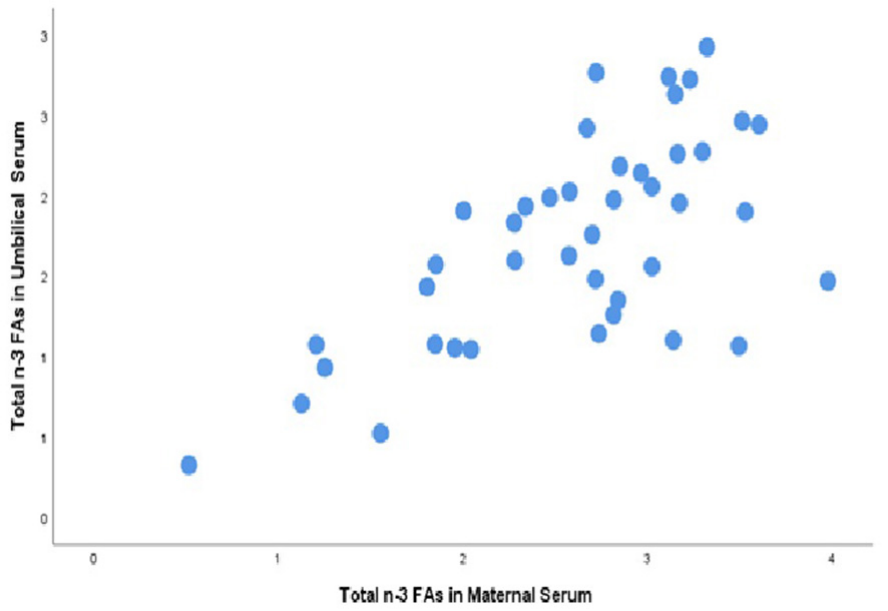
Non-parametric correlation between the concentration of total n-3 fatty acids in maternal venous serum and umbilical vein serum (ρ = 0.721; *P* = 0.001).

The particularly strong correlation for n-6 PUFAs likely reflects the efficient placental transfer of linoleic acid via membrane-bound fatty acid transport proteins, including FATP1, FATP4, and FAT/CD36. This finding is consistent with previous studies by Larqué et al. (4) and Haggarty (14) who demonstrated preferential placental handling of essential n-6 fatty acids in healthy pregnancies. The strong n-3 PUFA correlation (ρ = 0.721) further supports the importance of maternal dietary intake in determining fetal DHA and EPA status, which are critical for neurodevelopment (Figure 5).

The scatter plot shows that the comparison of total n-3 fatty acids between maternal venous serum and umbilical venous serum yielded a high correlation coefficient (ρ = 0.721, *P* = 0.001).

In contrast, the moderate correlation for saturated fatty acids (ρ = 0.562, *P* < 0.001) suggests greater placental regulation and preferential *de novo* fetal synthesis. The placenta actively modulates SFA transfer to protect the fetus from excessive lipid exposure, while the fetal liver retains substantial capacity for endogenous SFA synthesis from carbohydrate precursors (Supplementary Table S4).

These differential correlation patterns underscore the selective nature of placental fatty acid transfer: essential fatty acids that cannot be synthesized *de novo* exhibit stronger maternal-fetal correlations than non-essential lipids. From a clinical perspective, these findings suggest that maternal dietary interventions may directly influence fetal fatty acid status, particularly for essential PUFAs.

## 4 Discussion

This study suggests that regional dietary patterns are associated with differences in fatty acid composition in maternal venous serum, placental tissue, and fetal circulation. In particular, pronounced regional differences were observed in long-chain polyunsaturated fatty acids (PUFAs), especially docosahexaenoic acid (DHA) and arachidonic acid (AA), which are critical for fetal neurodevelopment and visual function (15–17).

Despite differences in traditional dietary habits, no significant regional differences were observed in maternal serum concentrations or proportions of saturated fatty acids. This finding likely reflects a general shift away from traditional diets and a widespread consumption of foods rich in saturated fats across Europe, as reported by Lauritzen et al. (18). Similar trends have been described in Mediterranean and non-Mediterranean populations, suggesting dietary homogenization driven by urbanization and food industrialization (19).

In contrast, monounsaturated fatty acids—particularly oleic acid—were more abundant in maternal serum from Split and Zagreb compared with Osijek. This pattern likely reflects higher olive oil consumption and partial adherence to Mediterranean dietary principles (20, 21). The presence of this pattern in Zagreb supports previous observations that migration and urban dietary habits contribute to the dissemination of Mediterranean dietary elements beyond coastal regions (22).

The most pronounced regional differences were observed in long-chain n-3 PUFAs. Maternal DHA concentrations and proportions were significantly lower in the Osijek group compared with Zagreb and Split, reflecting lower consumption of marine foods. These findings are consistent with studies showing that dietary intake of fish and seafood is the primary determinant of maternal DHA status during pregnancy (23, 24). Adequate maternal DHA levels are essential for optimal fetal brain and retinal development, particularly during the third trimester when DHA accretion is greatest (1, 15, 25).

Women from Osijek exhibited higher proportions of AA and total n-6 fatty acids, as well as a significantly higher AA:DHA ratio. Vegetable oils rich in linoleic acid, which dominate continental dietary patterns, are a major source of n-6 fatty acids and likely contribute to this imbalance (26–29). An elevated AA: DHA or n-6:n-3 ratio has been associated with adverse pregnancy outcomes, increased inflammatory signaling, and unfavorable cardiometabolic programming later in life (7, 30).

Fatty acid concentrations in umbilical venous serum strongly correlated with maternal serum levels but were consistently lower, confirming selective placental transfer. Importantly, the fetus exhibited higher relative proportions of DHA and AA and lower proportions of their precursors, supporting preferential placental transfer and/or fetal enrichment of biologically active long-chain PUFAs (31, 32). These findings align with previous evidence demonstrating active placental transport mechanisms favoring DHA and AA to meet fetal neurodevelopmental demands (14, 31, 32).

The detection of trans-fatty acids in maternal and fetal circulation, particularly in the Zagreb group, is of concern. Trans-fatty acids are known to interfere with PUFA metabolism by inhibiting desaturase and elongase activity and by competing with long-chain PUFAs for placental transport proteins (33–35). Experimental and observational studies have linked prenatal exposure to trans-fatty acids with reduced fetal DHA availability and impaired neurodevelopment (35). These findings underscore the importance of minimizing industrial trans-fat intake during pregnancy through targeted public health interventions. Elaidic acid is the predominant industrial trans fatty acid, primarily derived from partially hydrogenated vegetable oils commonly found in processed foods, margarines, and bakery products (33, 34). The presence of elaidic acid in umbilical venous serum confirms its transplacental transport to the fetus, consistent with previous reports demonstrating that trans fatty acids readily cross the placenta (35).

The higher elaidic acid concentrations observed in the Zagreb group may reflect greater consumption of industrially processed foods in urban settings compared to rural or coastal areas with more traditional dietary patterns. This finding is of clinical concern, as elevated maternal trans fatty acid levels have been associated with adverse pregnancy outcomes. Williams et al. reported that women with the highest levels of elaidic acid had a 7.4-fold increased risk of preeclampsia compared to those with the lowest levels (35). Furthermore, trans fatty acids are known to interfere with the metabolism of essential fatty acids by inhibiting delta-6 and delta-5 desaturase enzymes, potentially reducing the availability of DHA and arachidonic acid for fetal neurodevelopment (35–37).

Placental fatty acid composition partially reflected maternal dietary patterns, with higher DHA proportions observed in placentas from Split and Zagreb. However, direct correlations between individual maternal serum and placental fatty acids were not observed, likely due to complex placental metabolic, storage, and remodeling processes (36). Given the limited number of comparable studies and methodological heterogeneity, further research is needed to clarify placental fatty acid handling.

The regional dietary patterns observed in Croatia–coastal Mediterranean vs. European–parallel dietary contrast found in many other countries and regions worldwide. The Dalmatian coastal diet shares characteristic with traditional Mediterranean diets of Italy, Greece, and southern France, characterized by high fish and olive oil consumption. In contrast, the Slavonia continental diet resembles Central and Eastern European dietary patterns common in Hungary, Austria, Poland, and parts of Germany, with higher intakes of meat, dairy, and saturated fats. Thus, the observed associations between to regional diet and maternal-fetal fatty acid profiles are likely generalizable to populations with similar dietary dichotomies.

However, several factors may limit direct extrapolation to other populations. Differences in food preparation methods, specific fish species consumed, and available of fortified food may affect fatty acid bioavailability across populations.

From a clinical perspective, these findings have several practical implications:

First, prenatal nutritional counseling should be tailored to regional dietary contexts. Women from regions with traditionally low fish consumption may particularly benefit from education about the importance of DHA for fetal neurodevelopment and guidance on incorporating marine food sources or considering supplementation.

Second, the elevated AA:DHA ratio observed in the continental Slavonia population This study suggests that regional dietary patterns are associated with differences in fatty acid composition. Current international recommendations suggest 200–300 mg DHA daily during pregnancy (1), which may be difficult to achieve through diet alone in non-coastal regions.

Third, these findings provide a rationale for exploring region-specific public health strategies, including subsidizing fish products, implementing nutrition programs promoting Mediterranean dietary principles, and improving the availability of quality DHA supplements for pregnant women. Such strategies require evaluation in prospective intervention studies before clinical implementation.

Third, these findings provide a rationale for exploring region-specific public health strategies, including subsidizing fish products, implementing nutrition programs promoting Mediterranean dietary principles, and improving the availability of quality DHA supplements for pregnant women. Such strategies require evaluation in prospective intervention studies before clinical implementation. While not yet standard practice, point-of care fatty acid testing is becoming increasingly feasible.

These findings underscore the importance of minimizing industrial trans-fat intake during pregnancy through targeted dietary counseling and public health policies aimed at reducing trans fatty acids in the food supply.

### 4.1 Strengths and limitations of the study

A major strength of this study is its comprehensive assessment of fatty acid composition across three biologically relevant compartments—maternal venous serum, placental tissue, and umbilical venous serum. This integrated maternal–placental–fetal approach provides a more complete picture of fatty acid transfer and fetal exposure than studies limited to maternal blood alone. By simultaneously examining these compartments, the study offers valuable insight into selective placental transport and fetal enrichment of long-chain PUFAs, particularly DHA and AA.

Another important strength is the regional comparison within a relatively homogeneous population. By focusing on distinct Croatian regions with different dietary traditions (coastal vs. continental), the study isolates the impact of habitual dietary patterns while minimizing confounding by ethnicity, healthcare access, and genetic background. This strengthens the internal validity of the observed associations between diet-related fatty acid intake and maternal–fetal fatty acid status.

The use of direct biochemical measurements of fatty acids, rather than relying solely on dietary questionnaires, represents an additional strength. Objective quantification reduces recall bias and misreporting, which are common limitations of nutritional studies, and allows for a more accurate evaluation of biologically relevant fatty acid exposure. The inclusion of trans-fatty acid measurements further enhances the study's relevance from a public health perspective (38).

Several limitations should be considered when interpreting the findings.

First, the observational and cross-sectional design precludes causal inference. Although clear associations between regional dietary patterns and fatty acid profiles were identified, causality cannot be definitively established, and residual confounding by unmeasured lifestyle or socioeconomic factors remains possible.

Second, a fundamental limitation of this study is the absence of direct dietary intake assessment. No food frequency questionnaires, 24 h dietary recalls, or records of fish, olive oil, or supplement consumption were collected. Consequently, the observed regional differences in fatty acid profiles are consistent with known regional dietary patterns but cannot be causally attributed to specific dietary behaviors. All dietary interpretations should be regarded as hypothesis-generating and require confirmation in studies that include validated dietary assessment tools.

Third, the relatively small total sample size (*n* = 44) and unequal group sizes (Osijek *n* = 16, Zagreb *n* = 16, Split *n* = 12) represent important limitations that warrant discussion. The smaller Split group reflects practical recruitment constraints at that center during the study period. While *post-hoc* power analysis confirmed adequate statistical power (>95%) for detecting the large effect sizes observed for DHA (Cohen's d = 1.8), the study may have been underpowered to detect smaller but potentially meaningful differences in other fatty acids. The unequal group sizes may affect the precision of between-group comparisons, with wider confidence intervals for comparisons involving the Split group. These limitations underscore the exploratory nature of the study, and the findings should be interpreted as hypothesis-generating rather than definitive. Future confirmatory studies with larger, balanced sample sizes are warranted to validate these regional associations.

Fourth, fatty acid measurements were obtained at a single time point in late pregnancy. Fatty acid status changes dynamically throughout gestation, and longitudinal measurements would provide a more detailed understanding of temporal changes in maternal–fetal fatty acid transfer, particularly during earlier critical windows of fetal development.

Fifth, residual confounding by unmeasured variables–including maternal education, socioeconomic status, smoking status, physical activity, parity, and pre-pregnancy dietary habits–cannot be excluded. While the strict inclusion and exclusion criteria (normal BMI, normal OGTT, no lipid-lowering therapy, no n-3 fatty acid supplements, elective cesarean section only, absence of any pregnancy-related pathology) minimize several important sources of confounding, they cannot eliminate all. In particular, socioeconomic status and education level–which are known determinants of dietary quality–were not assessed. Future studies should incorporate comprehensive sociodemographic and lifestyle data to enable adjusted analyses.

The unexpectedly low oleic acid concentration observed in placental tissue from Osijek median 4.5 IQR 0.0–8.3 or and Zagreb groups median 4.7 IQR 0.0–6.2 (3.2 ± 3.1) warrant cautious interpretation. Some authors (39, 40) reported a higher proportion of oleic acid (9.1 ± 1.1%) in placental tissue compared to our findings.

Finally, the study did not assess functional neurodevelopmental or clinical outcomes in the offspring. While the observed differences in DHA, AA, and AA:DHA ratios are biologically meaningful and supported by existing literature, direct links to neonatal or long-term child outcomes could not be evaluated.

This exploratory study suggests that geographic region of residence in Croatia is associated with differences in maternal-fetal fatty acid composition. The sample size, lack of dietary questionnaires and cross-sectional design limit the strength of conclusions. These findings should be considered hypothesis-generating and require confirmation in larger studies with comprehensive dietary assessment. The strengths of the multi-compartment design and objective biochemical measurements outweigh the limitations, and the findings offer a solid foundation for future longitudinal and interventional studies aimed at optimizing maternal nutrition and fetal health.

## 5 Conclusions

From a nutritional perspective, this study indicates that regional dietary patterns are associated with differences in maternal, placental, and fetal fatty acid composition during pregnancy. Diets characterized by higher consumption of marine foods are associated with increased maternal and placental DHA levels and more favorable AA:DHA ratios, which are critical for optimal fetal neurodevelopment. The borderline-elevated AA:DHA ratio observed in pregnant women from Slavonia highlights the need for targeted nutritional counseling and, where appropriate, DHA and EPA supplementation. Additionally, the detection of trans-fatty acids in maternal and fetal circulation underscores the importance of reducing industrially processed foods during pregnancy. Overall, these findings suggest that maternal dietary fat quality may represent a modifiable factor influencing fetal fatty acid exposure, and support region-specific nutritional strategies to optimize pregnancy outcomes.

## Data Availability

The datasets presented in this study can be found in online repositories. The names of the repository/repositories and accession number(s) can be found in the article/ Supplementary material.
