# Supplementary material for: Regional dietary patterns and their impact on maternal, placental, and fetal fatty acid profiles during pregnancy: a multicenter study from Croatia
Source: Front Nutr. 2026 Aug 5;13:1883073. doi: 10.3389/fnut.2026.1883073 (PMC13485552; doi:10.3389/fnut.2026.1883073)
Supplement: Supplementary file 1 [file Table_1.docx]

Supplementary Material

# 1 Supplementary Data

**Table S1.** Total cholesterol esters, phospholipids, free fatty acids, and triglycerides in maternal venous serum

| Lipid fractions | Osijek (n=16)  Median and IQR | Zagreb (n=16)  Median and  IQR | Split (n=12)  Median and IQR |
| --- | --- | --- | --- |
| Cholesterol esters(µg/mL) | 92.8  (61.0-206.1) | 82.7  (66.5-117.9) | 38.0  (29.1-83.4) |
| Phospholipids (µg/mL) | 139.6  (41.4-25.0) | 237.3  (131.9-327.0) | 78.4  (50.6-139.6) |
| Free fatty acids (µg/mL) | 51.3  (16.3-65.6) | 52.1  (44.2-67.4) | 21.3  (7.2-56.2) |
| Triglycerides (µg/mL) | 49.8  (32.7-165.7) | 175.5  (70.1-277.7) | 109.1  (28.1-136.3) |

**Table S2.** Total cholesterol esters, phospholipids, free fatty acids, and triglycerides in placental tissue

| Cholesterol esters(µg/mL) | 21.4  (8.9-29.6) | 25.6  (14.7-31.2) | 45.7  (9.0-243.7) |
| --- | --- | --- | --- |
| Phospholipids (µg/mL) | 35.5  (12.0-43.2) | 168.0  (100.2-378.1) | 45.7  (21.3-125.4) |
| Free fatty acids (µg/mL) | 65.7  (19.5-134.0) | 101.6  (14.6-151.7) | 89.2  (47.7-248.9) |
| Triglycerides (µg/mL) | 8.3  (2.1-11.2) | 21.9  (17.2-26.6) | 29.9  (6.1-235.7) |

**Table S3.** Concentration of total fatty acids and concentration and proportion of saturated, monounsaturated, and polyunsaturated fatty acids in placental tissue

| Fatty acids | Osijek (n=16)  Median and IQR | Zagreb (n=16)  Median and IQR | Split (n=12)  Median and IQR | *P** | *Post hoc* |
| --- | --- | --- | --- | --- | --- |
| Total fatty acids (µg/mL) | 423.2  (201.5-588.5) | 227.6  (162.1-300.4) | 228.6  (140.5-305.1 | 0.164 |  |
| Palmitic acid (C16:0) (µg/mL) | 0.7  (0.4-1.4) | 0.6  (0.0-0.8) | 0.6  (0.3-0.9) | 0.751 |  |
| Stearic acid (C18:0) (µg/mL) | 51.9  (34.7-232.9) | 41.1  (20.0-80.5) | 30.3  (23.1-63.7) | **0.007** | O>S, O>Z |
| Total saturated fatty acids (µg/mL) | 252.2  (94.4-432.7) | 67.7  (45.7-87.3) | 72.7  (39.3-152.4) | **0.014** | O>Z, S |
| Palmitic acid (C16:0) (weight %) | 0.6  (0.1-0.9) | 0.6  (0.0-0.8) | 0.5  (0.3-0.9) | 0.181 |  |
| Stearic acid (C18:0) (weight %) | 19.9  (15.0-22.9) | 19.7  (12.9-27.0) | 23.2  (17.2-32.2) | 0.298 |  |
| Total saturated fatty acids (weight %) | 51.8  (46.5-59.0) | 36.7  (32.7-38.1) | 39.0  (34.8-60.5) | **0.006** | O>Z, S |
| Palmitoleic acid (C16:1 n-7) (µg/mL) | 1.3  (0.0-6.9) | 0.0  (0.0-0.6) | 0.,7  (0.0-1.1) | **0.015** | O>Z, S |
| Oleic acid (C18:1 n-9) (µg/mL) | 25.0  (0.0-30.2) | 0.0  (0.0-6.0) | 4.5  (0.0-8.3) | **0.003** | O>Z, S |
| Elaidic acid (C:18:1n-9 trans (µg/mL) | 13.1  (0.7-47.8) | 0.0  (0.0-3.7) | 0.0  (0.0-11.2) | **0.002** | O>S, Z |
| Total monounsaturated fatty acids (µg/mL) | 37.6  (17.0-66.3) | 31.9  (16.8-49.6) | 14.4  (10.0-27.0) | **0.168** | O>Z, S |
| Palmitoleic acid (C16:1 n-7) (weight %) | 0.4  (0.0-1.0) | 0.0  (0.0-0.7) | 0.7  (0.0-0.8) | **0.018** | Z>S, O |
| Oleic acid (C18:1 n-9) (weight %) | 4.9  (0.7-9.7) | 0.0  (0.0-6.0) | 4.1  (0.0-5.8) | **0.011** | O>S, Z |
| Elaidic acid (C:18:1n-9 trans  (weight %) | 2.5  (1.5-8.7) | 0.0  (0-2.7) | 5.8  (0.0-6.7) | 0.054 |  |
| Total monounsaturated fatty acids (weight %) | 12.5  (10.9-13.4) | 7.0  (2.9-11.8) | 12.4  (4.2-16.3) | **0.047** | O>Z, S |
| α-linolenic acid (C18:3 n-3) (µg/mL) | In trace | In trace | In trace | - |  |
| Eicosapentaenoic acid (C20:5 n-3) (µg/mL) | 0.0  (0.0-0.0) | 0.0  (0.0-6.9) | 0.0  (0.0-3.5) | 0.595 |  |
| Docosahexaenoic acid (C22:6 n-3) (µg/mL) | 6.8  (1.5-13.0) | 6.2  (5.3-16.3) | 4.1  (2.9-7.1) | 0.056 |  |
| Total polyunsaturated n-3 fatty acids) (µg/mL) | 9.5  (1.6-16.4) | 14.2  (8.6-18.5) | 7.6  (4.6-20.4) | 0.274 |  |
| α-linolenic acid (C18:3 n-3) (weight %) | - | - | - | - |  |
| Eicosapentaenoic acid (C20:5 n-3) (weight %) | 0.0  (0.0-0.0) | 0.0  (0.0-4.1) | 0.0  (0.0-2.0) | 0.427 |  |
| Docosahexaenoic acid (C22:6 n-3) (weight %) | 1.7  (0.6-3.1) | 2.7  (0.9-10.4) | 4.7  (1.4-8.7) | **0.015** | O<S, Z |
| Total polyunsaturated n-3 fatty acids (weight %) | 3.3  (1.4-6.5) | 8.7  (4.9-12.4) | 11.3  (3.3-11.4) | **0.013** | O<Z, S |
| Linoleic acid (C18:2 n-6) (µg/mL) | 10.2  (1.6-138.6) | 30.3  (0.8-47.9) | 1.3  (1.1-35.8) | **0.030** | O>S, Z |
| γ-linolenic acid (C18:3 n-6) (µg/mL) | 9.3  (5.6-19.3) | 28.8  (0.0-29.5) | 30.6  (26.3-35.6) | 0.062 |  |
| Arachidonic acid (C20:4 n-6) (µg/mL) | 62.9  (0.0-285.2) | 42.5  (23.0-78.7) | 36.2  (0.0-63.2) | **0.048** | O>S, Z |
| Total polyunsaturated n-6 fatty acids (µg/mL) | 149.2  (61.8-493.2) | 118.0  (88.7-131.7) | 105.8  (72.3-136.3) | 0.500 |  |
| Linoleic acid (C18:2 n-6) (weight %) | 8.7  (1.4-10.0) | 7.1  (0.5-23.6) | 1.4  (1.2-16.9) | 0.273 |  |
| γ-linolenic acid (C18:3 n-6) (weight %) | 0.7  (0.5-6.6) | 12.1  (0.0-16.2) | 14.6  (9.7-31.1) | 0.079 |  |
| Arachidonic acid (C20:4 n-6) (weight %) | 9.6  (1.5-18.6) | 2.0  (0,0-3.0) | 2.9  (0.0-10.6) | 0.098 |  |
| Total polyunsaturated n-6 fatty acids (weight %) | 32.8  (14.7-29.4) | 41,2  (19.2-51.7) | 41.2  (34.0-50.7) | 0.087 |  |

Kruskal-Wallis test: O=Osijek, Z=Zagreb, S=Split *Significance value adjusted by the Bonferroni correction for multiple tests

**Table S4.** Nonparametric (Spearman) correlations between fatty acids in maternal venous serum and umbilical venous serum.

|  | **Correlation between FAs in maternal vein serum and FAs in umbilical vein serum** | **Spearman ρ** | ***P*** |
| --- | --- | --- | --- |
| 1. | ln total fatty acid concentration in maternal venous serum vs. ln total fatty acid concentration in umbilical venous serum | 0.801 | 0.001 |
|  | ln total saturated fatty acid concentration in maternal venous serum vs. ln total saturated fatty acid concentration in umbilical venous serum | 0.562 | 0.001 |
| 2. | ln total monounsaturated fatty acid concentration in maternal venous serum vs. ln total monounsaturated fatty acid concentration in umbilical venous serum | 0.735 | 0.001 |
| 3. | ln total n-6 polyunsaturated fatty acid concentration in maternal venous serum vs. ln total n-6 polyunsaturated fatty acid concentration in umbilical venous serum | 0.808 | 0.001 |
| 4. | ln total n-3 polyunsaturated fatty acid concentration in maternal venous serum vs. ln total n-3 polyunsaturated fatty acid concentration in umbilical venous serum | 0.721 | 0.001 |
