# Supplementary material for: Regional dietary patterns and their impact on maternal, placental, and fetal fatty acid profiles during pregnancy: a multicenter study from Croatia
Source: Front Nutr. 2026 Aug 5;13:1883073. doi: 10.3389/fnut.2026.1883073 (PMC13485552; doi:10.3389/fnut.2026.1883073)
Supplement: Supplementary file 2 [file Table_2.docx]

**Table S2.** Total cholesterol esters, phospholipids, free fatty acids, and triglycerides in placental tissue

| Cholesterol esters(µg/mL) | 21.4  (8.9-29.6) | 25.6  (14.7-31.2) | 45.7  (9.0-243.7) |
| --- | --- | --- | --- |
| Phospholipids (µg/mL) | 35.5  (12.0-43.2) | 168.0  (100.2-378.1) | 45.7  (21.3-125.4) |
| Free fatty acids (µg/mL) | 65.7  (19.5-134.0) | 101.6  (14.6-151.7) | 89.2  (47.7-248.9) |
| Triglycerides (µg/mL) | 8.3  (2.1-11.2) | 21.9  (17.2-26.6) | 29.9  (6.1-235.7) |
