# Supplementary material for: Regional dietary patterns and their impact on maternal, placental, and fetal fatty acid profiles during pregnancy: a multicenter study from Croatia
Source: Front Nutr. 2026 Aug 5;13:1883073. doi: 10.3389/fnut.2026.1883073 (PMC13485552; doi:10.3389/fnut.2026.1883073)
Supplement: Supplementary file 3 [file Table_3.docx]

**Table S3.** Concentration of total fatty acids and concentration and proportion of saturated, monounsaturated, and polyunsaturated fatty acids in placental tissue

| Fatty acids | Osijek (n=16)  Median and IQR | Zagreb (n=16)  Median and IQR | Split (n=12)  Median and IQR | *P* | **P* |
| --- | --- | --- | --- | --- | --- |
| Total fatty acids (µg/mL) | 423.2  (201.5-588.5) | 227.6  (162.1-300.4) | 228.6  (140.5-305.1 | 0.164 |  |
| Palmitic acid (C16:0) (µg/mL) | 0.7  (0.4-1.4) | 0.6  (0.0-0.8) | 0.6  (0.3-0.9) | 0.751 |  |
| Stearic acid (C18:0) (µg/mL) | 51.9^a^  (34.7-232.9) | 41.1^a^  (20.0-80.5) | 30.3  (23.1-63.7) | 0.110 |  |
| Total saturated fatty acids (µg/mL) | 252.2^a^  (94.4-432.7) | 67.7^a^  (45.7-87.3) | 72.7  (39.3-152.4) | 0.014 | 0.020^a^ |
| Palmitic acid (C16:0)  (weight%) | 0.6  (0.1-0.9) | 0.6  (0.0-0.8) | 0.5  (0.3-0.9) | 0.181 |  |
| Stearic acid (C18:0) (weight %) | 19.9  (15.0-22.9) | 19.7  (12.9-27.0) | 23.2  (17.2-32.2) | 0.298 |  |
| Total saturated fatty acids (weight %) | 51.8^a^  (46.5-59.0) | 36.7^a^  (32.7-38.1) | 39.0  (34.8-60.5) | 0.006 | 0.006^a^ |
| Palmitoleic acid (C16:1 n-7) (µg/mL) | 1.3^a^  (0.0-6.9) | 0.0^a^  (0.0-0.6) | 0.7  (0.0-1.1) | 0.045 | 0.040^a^ |
| Oleic acid (C18:1 n-9) (µg/mL) | 4.5  (0.0-8.3) | 4.7^a^  (0.0-6.2) | 25.0^a^  (0.0-30.2) | 0.039 | 0.048 |
| Elaidic acid (C:18:1n-9 trans (µg/mL) | 0.0a  (0.0-3.7) | 13.1a  (0.7-47.8) | 0.0  (0.0-11.2) | 0.003 | 0.003 |
| Total monounsaturated fatty acids (µg/mL) | 14.4  (10.0-27.0) | 31.9  (16.8-49.6) | 37.6  (17.0-66.3) | 0.168 |  |
| Palmitoleic acid (C16:1 n-7) (weight %) | 0.4  (0.0-1.0) | 0.0  (0.0-0.7) | 0.7  (0.0-0.8) | 0.089 |  |
| Oleic acid (C18:1 n-9) (weight %) | 0.0a^b^  (0.0-6.0) | 4.1^a^  (0.0-5.8) | 4.9^b^  (0.7-9.7) | 0.018 | 0.048^a^  0.039^b^ |
| Elaidic acid (C:18:1n-9 trans  (weight %) | 2.5  (1.5-8.7) | 5.8  (0.0-6.7) | 0.0  (0-2.7) | 0.054 |  |
| Total monounsaturated fatty acids (weight %) | 7.0a  (2.9-11.8) | 12.4a  (4.2-16.3) | 12.5  (10.9-13.4) | 0.042 | 0.047 |
| α-linolenic acid (C18:3 n-3) (µg/mL) | In trace | In trace | In trace | - |  |
| Eicosapentaenoic acid (C20:5 n-3) (µg/mL) | 0.0  (0.0-0.0) | 0.0  (0.0-6.9) | 0.0  (0.0-3.5) | 0.595 |  |
| Docosahexaenoic acid (C22:6 n-3) (µg/mL) | 6.8  (1.5-13.0) | 6.2  (5.3-16.3) | 4.1  (2.9-7.1) | 0.056 |  |
| Total polyunsaturated n-3 fatty acids) (µg/mL) | 9.5  (1.6-16.4) | 14.2  (8.6-18.5) | 7.6  (4.6-20.4) | 0.274 |  |
| α-linolenic acid (C18:3 n-3) (weight %) | - | - | - | - |  |
| Eicosapentaenoic acid (C20:5 n-3) (weight %) | 0.0  (0.0-0.0) | 0.0  (0.0-4.1) | 0.0  (0.0-2.0) | 0.427 |  |
| Docosahexaenoic acid (C22:6 n-3) (weight %) | 1.7^a^  (0.6-3.1) | 2.7  (0.9-10.4) | 4.7^a^  (1.4-8.7) | 0.015 | 0.021a |
| Total polyunsaturated n-3 fatty acids (weight %) | 3.3^a^  (1.4-6.5) | 8.7  (4.9-12.4) | 11.3^a^  (3.3-11.4) | 0.013 | 0.019^a^ |
| Linoleic acid (C18:2 n-6) (µg/mL) | 10.2  (1.6-138.6) | 30.3  (0.8-47.9) | 1.3  (1.1-35.8) | 0.059 |  |
| γ-linolenic acid (C18:3 n-6) (µg/mL) | 9.3  (5.6-19.3) | 28.8  (0.0-29.5) | 30.6  (26.3-35.6) | 0.062 |  |
| Arachidonic acid (C20:4 n-6) (µg/mL) | 62.9^ab^  (0.0-285.2) | 42.5a  (23.0-78.7) | 36.2^b^  (0.0-63.2) | 0.001 | 0.007^a^  0.004^b^ |
| Total polyunsaturated n-6 fatty acids (µg/mL) | 149.2  (61.8-493.2) | 118.0  (88.7-131.7) | 105.8  (72.3-136.3) | 0.500 |  |
| Linoleic acid (C18:2 n-6) (weight %) | 8.7  (1.4-10.0) | 7.1  (0.5-23.6) | 1.4  (1.2-16.9) | 0.273 |  |
| γ-linolenic acid (C18:3 n-6) (weight %) | 0.7  (0.5-6.6) | 12.1  (0.0-16.2) | 14.6  (9.7-31.1) | 0.079 |  |
| Arachidonic acid (C20:4 n-6) (weight %) | 9.6  (1.5-18.6) | 2.0  (0,0-3.0) | 2.9  (0.0-10.6) | 0.098 |  |
| Total polyunsaturated n-6 fatty acids (weight %) | 32.8  (14.7-29.4) | 41,2  (19.2-51.7) | 41.2  (34.0-50.7) | 0.087 |  |

Kruskal-Wallis test, *Significance value adjusted by the Bonferroni correction for multiple tests
