# Supplementary material for: Regional dietary patterns and their impact on maternal, placental, and fetal fatty acid profiles during pregnancy: a multicenter study from Croatia
Source: Front Nutr. 2026 Aug 5;13:1883073. doi: 10.3389/fnut.2026.1883073 (PMC13485552; doi:10.3389/fnut.2026.1883073)
Supplement: Supplementary file 4 [file Table_4.docx]

**Table S4.** Nonparametric (Spearman) correlations between fatty acids in maternal venous serum and umbilical venous serum.

|  | **Correlation between FAs in maternal vein serum and FAs in umbilical vein serum** | **Spearman ρ** | ***P*** |
| --- | --- | --- | --- |
| 1. | ln total fatty acid concentration in maternal venous serum vs. ln total fatty acid concentration in umbilical venous serum | 0.801 | 0.001 |
|  | ln total saturated fatty acid concentration in maternal venous serum vs. ln total saturated fatty acid concentration in umbilical venous serum | 0.562 | 0.001 |
| 2. | ln total monounsaturated fatty acid concentration in maternal venous serum vs. ln total monounsaturated fatty acid concentration in umbilical venous serum | 0.735 | 0.001 |
| 3. | ln total n-6 polyunsaturated fatty acid concentration in maternal venous serum vs. ln total n-6 polyunsaturated fatty acid concentration in umbilical venous serum | 0.808 | 0.001 |
| 4. | ln total n-3 polyunsaturated fatty acid concentration in maternal venous serum vs. ln total n-3 polyunsaturated fatty acid concentration in umbilical venous serum | 0.721 | 0.001 |
